# Evaluation on Phantoms of the Feasibility of a Smart Bra to Detect Breast Cancer in Young Adults

**DOI:** 10.3390/s19245491

**Published:** 2019-12-12

**Authors:** Marie-Valérie Moreno, Edouard Herrera

**Affiliations:** Research and Development Center, RunSys, 162 Lot le verger, 69380 Chasselay, France; edouard.herrera@runsys.eu

**Keywords:** breast cancer, breast density, electrical characteristics, smart bra

## Abstract

Breast cancer is the most common cancer observed in women. Although mammography is a recognized method, it remains ionizing and cannot be used routinely or in young adults, leaving up to two years between two diagnoses. Prior to validation on human subjects, this study aims to validate on phantoms the feasibility of quantifying breast density and detecting breast cancer tumors using a smart bra in young women. Six phantoms with various densities and seven phantoms with various volumes of modelized tumor were prepared and measured with a smart bra, including an electrophysiological module. There was a significant difference between the “healthy phantoms” and the “tumor phantoms” with *P*(Student) = 0.008 (Shapiro–Wilk *p* = 0.846, samples follow a normal distribution; Fisher variance test, *p* = 0.287). In addition, this study seems to indicate the possibility of discriminating various types of tumorous and healthy breast tissue using a smart bra, in high density breast. However, a new study on a large sample of human subjects will be required to generate new models, including resistive, capacitive, and other sensor parameters versus reference data collected from imaging.

## 1. Introduction

Breast cancer is the most common cancer observed in women in France, as well as in the European Union and the United States. The number of cases observed each year has tended to decrease since 2005, yet this disease remained as the leading cause of cancer death among women in 2012. If detected at an early stage, this cancer can be cured in 9 out of 10 cases.

According to the National Cancer Institute [1], there were 48,763 new cases estimated in 2012, 11,886 of which resulted in the death of the patient. In 8 out of 10 cases, these cancers affected women aged 50 and over.

Breast cancer screening mainly uses mammography (breast X-rays under different views). This technique can lead to overdiagnosis; some women are found to have precancerous lesions that have never developed. According to the National Institut of Cancer (INCa), the rate of overdiagnosis is estimated at 10% to 20%, or 2 to 3 cases of overdiagnosis for each death prevented.

In addition, this technique uses X-rays, which have a certain carcinogenic effect. In women over 50 years of age, mammography-induced cancers are estimated to cause 1 to 5 deaths per 100,000 patients examined. The risk is higher before this age, as the breasts are denser and the examination requires higher doses of X-rays.

Before the age of 50, a mammography would be more harmful than beneficial. However, in France, 77% of women have already had several mammograms before this age.

During the Days of the French Society of Senology and Breast Pathology [2], the problem of interval cancers was finally raised. Its rate of 18% remains significant. It appears that implementing more frequent screenings (every year, for example) is not the solution because it would increase the rate of false positives as well as the irradiation of women. Between 54% and 60% of interval cancers are new tumors that could not be detected by mammography. The others, however, are the result of errors, which can be technical (about 20% of cases) or human (about 30% of cases).

**Figure 1:**
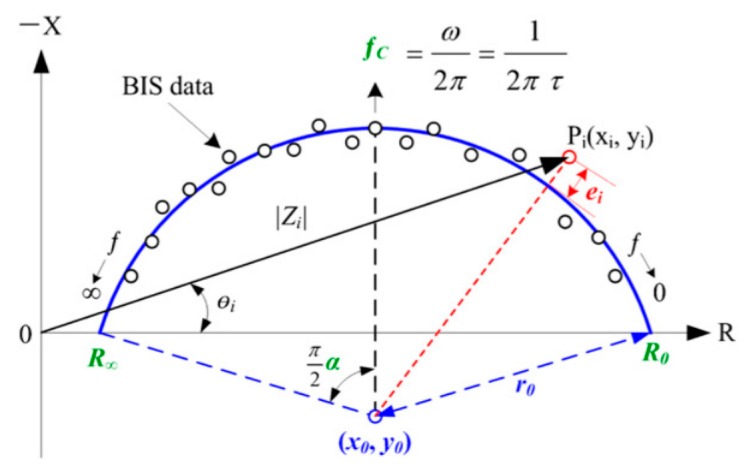
Description of the Cole–Cole model.

Many projects have been carried out over the past twenty years to overcome these problems. Ng et al. [3] reviewed the various technical studies and solutions that have been developed, particularly for the diagnosis of breast cancer, by exploring the electrical characteristics of tissues. More specifically, Du [4], Gupta [5], Stojadinovic [6], Jossinet [7], and other authors [8] focused on detecting breast cancer using bioelectrical impedance analysis (BIA), demonstrating up to 82.62% sensitivity and 95.79% specificity in the discrimination of benign and malignant breast tumors based on simple BIA models (injection of a low current and analysis of the induced voltage [9]) using characteristic tissue frequency (fc, kHz), which is the extrapolation of resistances using the Cole–Cole model (shown in Figure 1) at zero frequency and infinite frequency, known as Re (ohms) and Rinf (ohms), respectively [10].

Other parameters, derived from the same model, can enrich the equations:

- *C* (*xc*, *yc*) (center of the Cole–Cole circle, *r*: radius of the Cole–Cole circle)
- *Rc*, *Xc*, and *Fc* data, known as characteristic curvature data (characteristic resistance, characteristic reactance and characteristic frequency)
- *Ri* (modelled resistance of the intracellular zone in ohms)
  (1) $$R_{i}=\frac{R_{e}R_{\infty }}{R_{e}-R_{\infty }}$$
- Alpha *α* (the phase angle in degrees at the characteristic frequency)
  (2) $$\alpha =A\tan\left(\frac{yc+r}{xc}\right)*\frac{180}{pi}$$
- Tau *τ* (the ion relaxation time in µs)
  (3) τ=(Re+Ri)∗Cm
  where *Cm* is the capacity of the cell membrane in Farad, as follows:
  (4) $$Cm=\frac{1}{2\pi Xc}=\frac{1}{2\pi \left(yc+r\right)}$$

Estrela da Silva et al. [11] have shown that it is possible to differentiate between certain types of healthy or pathological breast tissue (connective tissue, fat tissue, glandular tissue, carcinoma, fibroadenoma, and mastopathy) by studying certain complementary parameters, including the area under the impedance circle curve, its length, and the distance between the extreme points.

Along with these direct technologies, which seek to characterize or detect breast tumors, two other indirect methods are used to estimate cancer development: breast density qualification and thermography.

Breast density is an established risk factor for breast cancer, as shown in Table 1 [12,13].

This is clinically established by tactile methods, imaging, and other methods [14]. It is also one of the factors of “noise”, as it varies based on the menstrual cycle [15].

White et al. [16] analyzed 2591 women aged 40 to 49 years, without hormone therapy, and classified 24% of breasts as type 4 during the first week of the cycle and 23% during the second week, while for weeks three and four the percentage was 28%, which is a significant variation (*p* = 0.04) regardless of body mass index (BMI). They used the BI-RADS (Breast Imaging-Reporting and Data System) classification to define breast typology:

- Stage/type 1: the breast is almost entirely fat (homogeneous fat) (less than 25% of the mammary gland).
- Stage/type 2: there are scattered fibro-glandular opacities (heterogeneous fat) (approximately 25 to 50% of the gland).
- Stage/type 3: Breast tissue is dense and heterogeneous (heterogeneous density), making it difficult to detect small masses (approximately 51% to 75% of the gland).
- Stage/type 4: the breast tissue is extremely dense (homogeneous density). This can reduce the sensitivity of mammography (>75% of the gland).

In addition, Renaud et al. [17], in a European epidemiological study of a cohort of 40,293 women aged 50 to 67 years, highlighted the evolution of breast density with age, as shown in Table 2, with a higher propensity for dense breasts in younger women, and therefore a greater difficulty in detecting the presence of a tumor.

A simple, non-operator-dependent method to quantify breast density could help to diagnose breast cancer as well as to qualify natural “noise” due to the variation in density during the menstrual cycle, which affects tumor detection.

Finally, skin thermography is another indirect indicator correlated with the detection of breast cancer. Similar to breast density, it can also vary during the hormonal cycle. A warmer area (red in clinical thermography), especially between 2 breasts in the same place, may show an increase in vascularization and more particularly inflammation around the tumor. However, a variation in temperature is not necessarily due to the presence of a tumor.

Integrating these various parameters into one built-in device, such as a bra, should make it possible to generate breast cancer detection scores showing better specificity and sensitivity.

The overall objective of the project is, therefore, to validate a connected breast cancer diagnostic bra that will allow:

- Early diagnosis at any age using non-ionizing technologies,
- Limitation of interval cancers as well as overdiagnosis by daily monitoring and considering physiological “noise”.

Prior to validation on human subjects, this study aims to validate, on phantoms, the feasibility of quantifying breast density and detecting breast cancer tumors using a smart bra.

## 2. Materials and Methods

### 2.1. The Phantoms

The animal model, although relevant, remains cumbersome, and some electrical characteristics of small mammals, such as rodents, are far from similar to those of humans, particularly due to the difference in metabolism (the average heart rate of a rodent can range from 250 to 450 beats per minute [18]).

The representativity of human tissue in phantoms is also limited but they have the advantage of being more controllable and easier to implement.

Several studies describe the development of techniques and ingredients to prepare materials that mimic human tissues [19]. The most commonly used materials include water-based, agarose, gelatin, and gel materials. Phantoms based on agarose and gelatin (also known as hydrogels) are the most commonly used alternatives. Agarose provides consistency, while NaCl increases conductivity.

Although these phantoms are stable for several weeks [20], microbial development can occur, distorting measurements. In addition, a precise preparation procedure must be followed to optimize the reliability of the measurements, optimizing for cooking time, order of integration and method of integration of the ingredients, refrigeration time, rest time at room temperature before measurements, etc. [21,22].

Jossinet [23], in 1996, presented a study on the electrical characteristics of in vitro tissues, including mammary glandular tissues, connective tissues, adipose tissues, and tissues with mastopathy, carcinomas, and fibroadenomas. These studies did not converge with those of other authors regarding the capacitive behavior of healthy and cancerous tissues. They do, however, agree on the resistive and conductive characteristics of the tissues, according to Table 3.

There is increased conductivity (a decrease in resistivity) in the tissue surrounding the tumor, which is to be expected given the inflammation of the extracellular compartment around the tumor. Since the intrinsic characteristics of tumor cell membranes remain uncertain, it is difficult to predict whether the electrons access the internal characteristics of tumors. In these initial tests, we will, therefore, focus on the detection of the equivalent of peripheral tissues, assuming a coefficient of 10 between the resistivities of the 2 types of tissues.

Phantoms hemispheres are 11.5 cm in diameter. Table 4 presents the compositions of the different types of phantoms.

Table 5 and Table 6 present the phantoms that were included in the study. Some phantoms had cracks or other defects that did not allow them to be included. The aim of the device is to supplement current methods, particularly mammography. The latter method presents diagnostic difficulties in the case of dense breasts, especially in young adults. At this preliminary stage of preparation of the clinical study, we wanted to test the worst clinical case via “tumor” phantoms based on breasts of maximal density.

**Figure 2:**
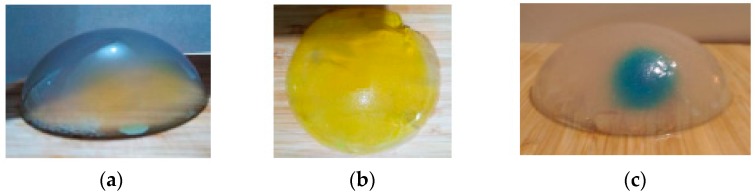
Photos of the phantoms studied: (**a**) F4 “breast density” phantom, (**b**) F1 “breast density” phantom, and (**c**) F2 “tumor” phantom.

Figure 2 shows photos of the phantom F4 (dense breast with only 30% adipose tissue), F1 (very low-density breast with 80% adipose tissue), and F2′ (breast with a 16.5 cm^3^ tumor).

### 2.2. The Device

**Figure 3:**
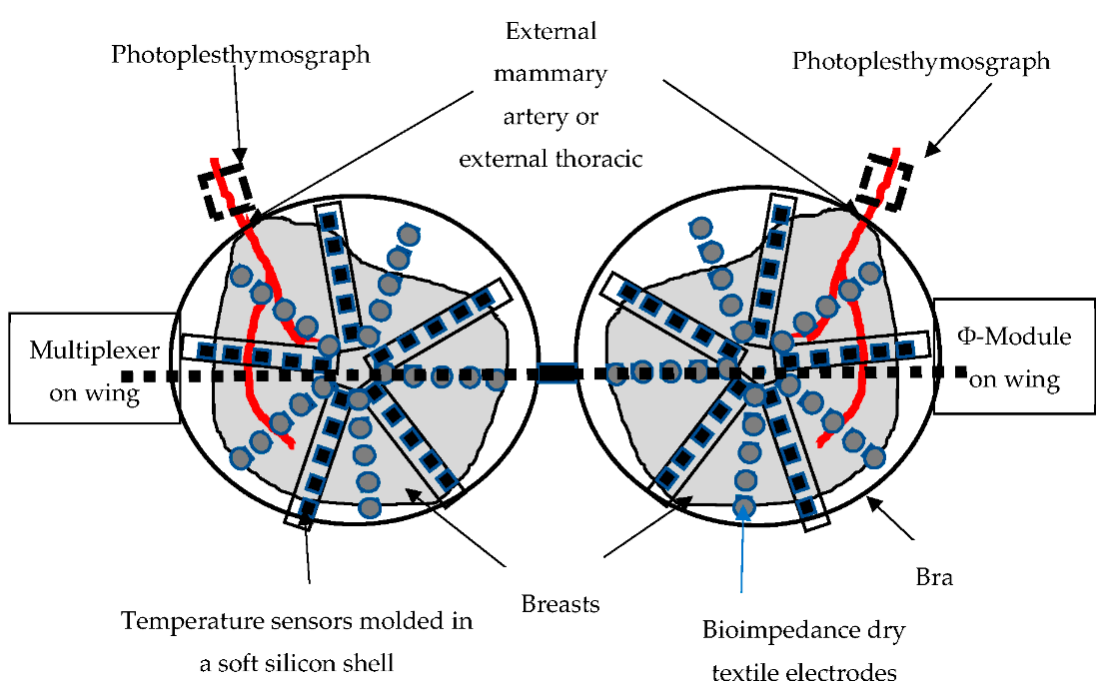
Schematic bra prototype under development (all the cables connecting the electrodes, the temperature sensors, and the PPG to the multiplexer are molded in the silicone shell placed between the double layer of the textile, forming the cap).

The bra prototype designed for the clinical study (Figure 3), which is still under development, is based on three types of sensors: temperature (breast thermography), photoplethysmograph (PPG) (oxygen saturation of the external thoracic artery), and bioimpedance of the tissue. In total, 120 temperature measurement points (60 per breast) are organized in stars for each breast. These sensors are managed via a multiplexer connected to the electrophysiological module via a USB link. The latter is also connected to two optical PPG sensors, each based on two green LEDs and a photodiode (sample rate 100 Hz, pulse width 115,2 ms) placed on the arterial vascularization of each breast. Finally, 120 electrodes (60 per breast) knitted in silver wire are organized in stars on each breast for the acquisition of the electrical parameters via a multiplexer connected to the electronic module. The module is driven by an application on an android tablet (Android 9.0, API (Application Programming Interface) Level 28).

**Figure 4:**
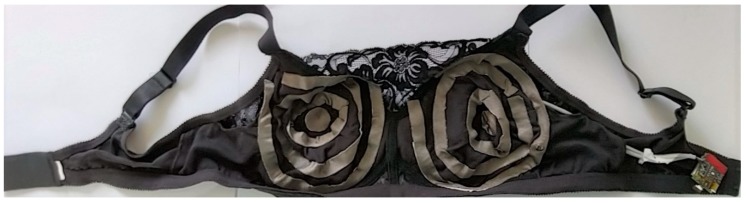
Photo of the “simplest” prototype device (without temperature and estimation of arterial oxygen saturation (SPO_2_) sensors).

In this study, our aim is to first explore the electrical parameters on phantoms that are unable to represent the complexity of human tissue. Then, we simplify the device for this purpose, as shown in Figure 4.

The textile electrodes were replaced by metal electrodes in order not to risk a modification of the characteristics of the electrodes during the experiments by the gel absorption of the phantoms in the tissue. Only the right bra cup was used. Four electrodes (10 mm size) were combined with the electrophysiological module according to a quadripolar measurement using ϕ-module (ZRange (0–2500 ohms); Zreal CV% 0.025% and Zimg CV% 0.408%; accuracy (all frequencies) Zr mean error 2.60% ± 2.01% and Zimg mean error 0.43% ± 0.84%) from RunSys, France.

The device was tested first on the universal Resistance//Resistance-Capacitor phantom (680 ohms//910 ohms - 2.7 nF). We obtained Zr mean error of 2.97% ± 1.15% and Zimg mean error of 0.5% ± 0.80%. The CV% obtained was closely to that of the module alone.

A current of 32 µA (at low frequency <8 KHz with high-pass filters (HPF) and low-pass filters (LPF) in bypass, 20 *v*/*v* amplifier, 64 sample per second (sampling frequency)) was injected and the real parts of the Zr impedances (ohms) of the different phantoms were collected.

### 2.3. The Algorithms

Using equations (5 to 9), the device estimated the breast density percentage and a tumor risk score (developed on a subpopulation of the sample (F1′, F3′, F4′, F5′, F4, F5, F6) and validated on the remaining subpopulation (F6′, F7′, F8′, F1, F2, F3)).

The breast density is estimated by a multivariable linear regression, calculated as Equation (5):

(5) Breast Density=a+bx1+cx2+⋯+ixi

where *a*, *b*, …, *i* are constants and *x*1, *x*2, …, *xi* are experimental variables of the equation.

The tumor risk score is estimated by a binomial law, calculated as Equation (6):

(6) Tumor Risk Score=1(1+e(−(a+bx1+cx2+⋯+ixi)))

where *a*, *b*, …, *i* are constants and *x*1, *x*2, …, *xi* are experimental variables of the equation.

In this study, having fewer experimental variables, we used the simplified logistic binomial law (two parameters µ, s), with its density function calculated as Equation (7):

(7) f(x;µ, s)=e−((x−µ)/s)s(1+e−(x−µs))²

Then, its distribution function is calculated as Equation (8):

(8) F(x;µ, s)=11+e−(x−µs)

We notice in our study the Esperance E(X) = µ=0 and the variance Var (X) = s²π²/3, inducing s = 1.

Then, the obtained distribution function of the tumor risk score is calculated as Equation (9):

(9) F(x)=11+e−(x)

with *x* corresponding to the electrical characteristics measured.

## 3. Results

### 3.1. Repeatability of Measurements

Table 7 shows average coefficients of variation of 0.69% ± 0.81% and 0.19% ± 0.08% for the “breast density” and “tumor” phantoms, respectively, thus ensuring that the repeatability of measurements is over 99%.

### 3.2. Raw Data

Table 8 shows the raw data obtained for each selected phantom.

### 3.3. Estimation of Breast Density Using the Device

**Figure 5:**
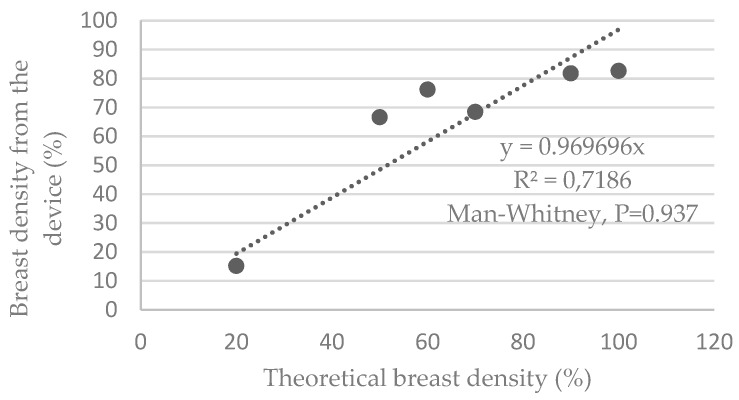
Graphs representing the breast density estimated by the device versus the theoretical density of the phantoms.

Figure 5 shows that the device underestimates the theoretical breast density by an average of 3.1%. The dispersion indicates a R^2^ value of 0.72, with a minimum deviation of −1.6% and a maximum deviation of −17.4%. 

The average deviation obtained is 0.009% ± 13.651%. The Shapiro–Wilk tests verified that the samples do not follow a normal distribution. This, therefore, led us to a nonparametric paired Mann–Whitney test, indicating *P* = 0.937.

### 3.4. Detection of the Presence of a Breast Tumor Using the Device

Table 9 describes the actual impedances measured on the different phantoms. An average of 19.3 ± 1.7 ohms is obtained for phantoms containing a tumor versus 24.9 ± 2.9 ohms for phantoms simulating healthy breasts, with an equivalent intrinsic density of 100% (or 0% adipose tissue), which are the most difficult conditions to discriminate from tumor tissue. The mean difference is −22.7% ± 6.8%. There is a significant difference between samples with *P* (Student) = 0.008 (Shapiro–Wilk *p* = 0.846, samples follow a normal distribution; Fisher variance test, *p* = 0.287). 

If we consider the sample comprising a set of healthy breasts with a variable density with the presence of simulated fat tissue from 0% to 80%, the significant mean difference is −30.6% ± 4.2% with *P* (Mann–Whitney) = 0.001 (Shapiro–Wilk *p* = 0.012, the samples do not follow a normal distribution); that is, 6.3% more than for very dense breasts.

**Figure 6:**
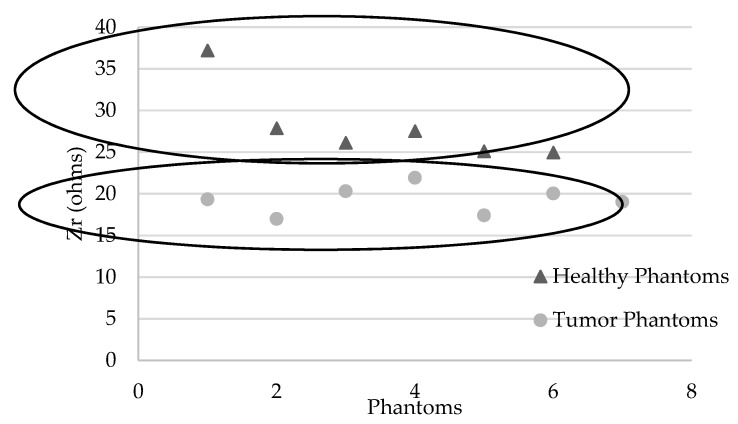
Graphs representing Zr (ohms) for each phantom: healthy (with various densities) and tumor phantoms (with various volumes of simulated tumor in high density phantoms).

Figure 6 shows two groups of data according to healthy or tumor phantoms.

As described in Table 10, a tumor risk score of 100% is recorded for phantoms containing a simulated tumor and 0% for “healthy” phantoms at different densities.

**Figure 7:**
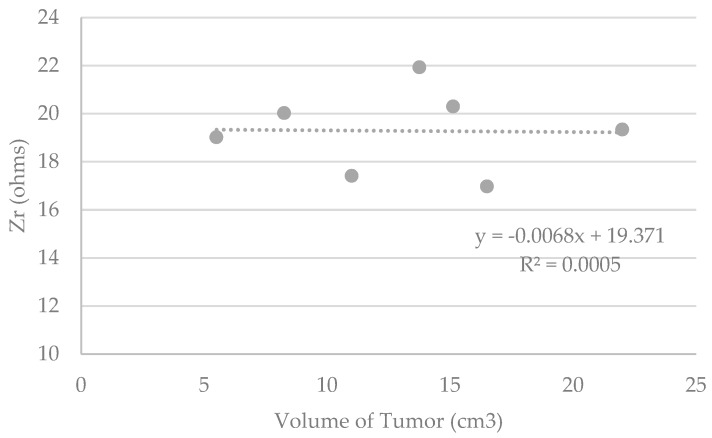
Graphs representing Zr (ohms) measured by the device versus the theorical volume of the tumor included in the phantoms (cm^3^).

Although it seems possible to discriminate healthy and diseased tissue modeled in phantoms, Figure 7 does not show any correlation between the measured data and the theoretical tumor volumes.

## 4. Discussion

It can be assumed that some significant differences between the device’s estimation of breast density and theoretical breast density are due to the sum of model-related errors, as well as to the fabrication of the phantoms. Indeed, the model used in this study is not robust enough due to the small population used to generate the model. In addition, incorporating a specific percentage of simulated fat tissue into a healthy tissue phantom is not easy. Theoretical breast densities are, therefore, biased.

Furthermore, since the capacitive characteristics of the tissues are discriminative, it can be assumed that the estimation of breast density based on the resistive and capacitive properties of the tissues making up the breast will be more accurate. In addition, these models will have to be generated and compared with imagery to ensure baseline data, and on larger populations to increase the robustness of the models.

As expected, the difference between healthy and tumorous tissues is negative, with the latter being more conductive (or less resistive) than healthy tissues, especially if they contain insulating adipose tissue. It would have been interesting to also test a population of phantoms with varying densities and including tumors, representing older women. We can imagine that there would be a risk of overlap between the data measured in a diseased breast with higher density, including a tumor, and those of a healthy breast of lower density; the soft tissue could be confused with the tumor tissue. This would be particularly true in the case of this type of phantom, not considering the capacitive or thermal differences of a healthy soft tissue versus a tumor tissue.

In addition, incorporating a specific volume of simulated tumoral tissue into a healthy tissue phantom is not easy. Theoretical tumoral volumes are, therefore, biased. This may explain in part why no correlation was found between the raw data and the tumor volumes. Moreover, given the choice of using phantoms of maximum density as a base for the inclusion of tumors, which are very conductive, one might think that the current would have an easily conductive path but would not fully cover the tumor volume. This should not occur in the case of complex human tissues with more resistive or capacitive parts, even in the case of very dense breasts with a high proportion of soft tissue.

The results of the tumor risk score have to be qualified. Phantoms do not simulate all the complexities of human tissue, either healthy or tumoral (variation of the temperature of the tumor zones, variation of the transmembrane exchanges impacting the capacitive characteristics of tissues, etc.).

Besides, the experimental conditions did not allow phantoms to be studied with tumors smaller than 5 cm^3^ and detecting tumors larger than 20 cm^3^ is of no clinical use. It is likely that it will be necessary to explore the capacitive properties of healthy and tumorous human tissues (more difficult to control on phantoms) in order to discriminate between low volumes.

## 5. Conclusions

However, the resistive models used indicate significant differences between the phantoms, which came from the models themselves (insufficient population), as well as from bias from the phantom.

A new study on a large sample of healthy human subjects will be required to generate new models, including resistive, capacitive and other sensor parameters versus reference data collected from imaging.

In addition, this study seems to indicate the possibility of discriminating various types of tumorous and healthy breast tissue using electrical methods in young adults. A new study on human subjects with various typologies (to test the level of specificity of the device) and various volumes (to test the level of sensitivity), including minimal tumors, is required to be able to validate the complete device. The latter will then be used in its entirety with its additional sensors (temperature, photoplethysmography, etc.). Further longitudinal studies can then be conducted to qualify the effects of the hormonal cycle and daily life on predictive models.

## 6. Patents

Patent FR 1,903,655 resulted from a part of this work.

## Figures and Tables

**Table 1:** Combined relative risks of breast cancer in the general population according to the classification used to assess breast density. RR = relative risk, CI95 = 95% confidence interval [13].

Classification	Incidental Studies RR[CI95]	Preliminary Studies RR[CI95]
Wolfe		
N1 (Adipose Breast)	1	1
P1 (<25% Glandular Density)	1.8 [1.4–2.2]	1.3 [1–1.5]
P2 (>25% Glandular Density)	3.1 [2.5–3.7]	2 [1.3–3]
DY (Dysplastic Breast)	4 [2.5–6.3]	2.4 [2,3]
Density (%)		
<5	1	1
5–24	1.8 [1.5–2.2]	1.4 [1.1–1.8]
25–49	2.1 [1.7–2.6]	2.2 [1.8–2.8]
50–74	2.9 [2.5–3.4]	2.9 [2.3–3.8]
>75	4.6 [3.6–5.9]	3.7 [2.7–5]
BI-RADS		
1 (<25% Glandular Density)	1	1
2 (25–50% Glandular Density)	2.2 [1.6–3]	1.6 [0.9–2.8]
3 (51–75% Glandular Density)	3 [2.2–4.1]	2.3 [1.3–4.3]
4 (>75% Glandular Density)	4 [2.8–5.7]	4.5 [1.9–10.6]

**Table 2:** Rates of mammographic morphological breast types at 50 to 67 years of age [17].

Type	50 Years Old	67 Years Old
1	14.5%	28%
2	43%	54%
3	38%	16%
4	5.6%	1.2%

**Table 3:** Resistive and conductive characteristics of breast tissue [23].

	Tumor Fabrics	Tissue Surrounding the Tumor	Healthy Breast Tissue
**⍴ (Ω.cm)**	250–500	125	1000

**Table 4:** Definition of phantom characteristics.

	Peripheral Tissues of the Tumor, Blue Color	Healthy Mammary Soft Tissues (Fibro-Glandular and Connective Tissue), Clear Color	Adipose Tissue, Yellow Color
**Composition**	4 g/L agarose 1 L demineralized water 10 g/L NaCl	4 g/L agarose 1 L demineralized water 1 g/L NaCl	4 g/L agarose 1 L demineralized water 0 g/L NaCl

**Table 5:** Characteristics of the “breast density” phantoms included in the study.

“Breast Density” Phantoms F	% of Healthy Soft Tissue Equivalent to Breast Density (Fibro-Glandular and Connective Tissue)	% of Adipose Tissue
F1	20	80
F2 (Breasts equivalent to those of healthy women in their 60s)	50	50
F3	60	40
F4	70	30
F5	90	10
F6 (Theoretical breasts of maximum density, without fat tissue)	100	0

**Table 6:** Characteristics of the “tumor” phantoms included in the study.

“Tumor” Phantoms F’	Volume (cm^3^) of Tumor Phantom Randomly Integrated into a 100% Density Phantom % of Healthy Soft Tissue
F1′	22.0
F2′	16.5
F3′	15.1
F4′	13.8
F5′	11.0
F6′	8.3
F7′	5.5

**Table 7:** Coefficient of variation (%) obtained for the different phantoms.

“Breast Density” Phantoms		F1	F2	F3	F4	F5	F6
Coefficient of Variation (%)		0.04	1.13	0.07	2.08	0.73	0.07
“Tumor” Phantoms	F1′	F2′	F3′	F4′	F5′	F6′	F7′
Coefficient of Variation (%)	0.15	0.15	0.26	0.29	0.12	0.23	0.10

**Table 8:** Raw data obtained for each phantom.

“Breast Density” Phantoms		F1	F2	F3	F4	F5	F6
Zr (ohms)		37.18	27.85	26.10	27.51	25.10	24.93
“Tumor” Phantoms	F1′	F2′	F3′	F4′	F5′	F6′	F7′
Zr (ohms)	19.33	16.97	20.30	21.92	17.41	20.02	19.02

**Table 9:** Real impedance (Zr, ohms), measured on phantoms, including tumors versus healthy phantoms (*, *p* < 0.05; **, *p* < 0.01; ***, *p* < 0.001).

	100% Low-Density Breasts with Tumor in Random Position (from 5 to 22 cm^3^)	100% Density Healthy Breasts	Healthy Breasts (Density Varies from 20% to 100%)
Zr Mean ± SD (Ohms)	19.3 ± 1.7	24.9 ± 2.9	28.1 ± 4.6
Difference Mean ± SD (%)		−22.7 ± 6.8**	−30.6 ± 4.2 ***

**Table 10:** Tumor risk score for each phantom in the validation subpopulation.

	Tumor Risk Score
**F6′**	**100%**
**F7′**	**100%**
**F8′**	**100%**
**F1**	**0%**
**F2**	**0%**
**F3**	**0%**
